# A novel approach to denoising ion trap tandem mass spectra

**DOI:** 10.1186/1477-5956-7-9

**Published:** 2009-03-17

**Authors:** Jiarui Ding, Jinhong Shi, Guy G Poirier, Fang-Xiang Wu

**Affiliations:** 1 Department of Mechanical Engineering, University of Saskatchewan, 57 Campus Dr., Saskatoon, SK S7N 5A9, Canada; 2 Division of Biomedical Engineering, University of Saskatchewan, 57 Campus Dr., Saskatoon, SK S7N 5A9, Canada; 3 Health and Environment Unit, Laval University Medical Research Center (CHUL), Faculty of Medicine 2705 Boul. Laurier, Quebec City, QC GIV 4G2, Canada

## Abstract

**Background:**

Mass spectrometers can produce a large number of tandem mass spectra. They are unfortunately noise-contaminated. Noises can affect the quality of tandem mass spectra and thus increase the false positives and false negatives in the peptide identification. Therefore, it is appealing to develop an approach to denoising tandem mass spectra.

**Results:**

We propose a novel approach to denoising tandem mass spectra. The proposed approach consists of two modules: spectral peak intensity adjustment and intensity local maximum extraction. In the spectral peak intensity adjustment module, we introduce five features to describe the quality of each peak. Based on these features, a score is calculated for each peak and is used to adjust its intensity. As a result, the intensity will be adjusted to a local maximum if a peak is a signal peak, and it will be decreased if the peak is a noisy one. The second module uses a morphological reconstruction filter to remove the peaks whose intensities are not the local maxima of the spectrum. Experiments have been conducted on two ion trap tandem mass spectral datasets: *ISB *and *TOV*. Experimental results show that our algorithm can remove about 69% of the peaks of a spectrum. At the same time, the number of spectra that can be identified by Mascot algorithm increases by 31.23% and 14.12% for the two tandem mass spectra datasets, respectively.

**Conclusion:**

The proposed denoising algorithm can be integrated into current popular peptide identification algorithms such as Mascot to improve the reliability of assigning peptides to spectra.

**Availability of the software:**

The software created from this work is available upon request.

## Background

Nowadays, two approaches are widely used for peptide identification from tandem mass (MS/MS) spectra: database searching and *de-novo *sequencing [1,2]. *De-novo *sequencing algorithms assign peptides to *MS/MS *spectra based on the spectra alone. Therefore, these algorithms are invaluable for the identification of both known and unknown peptides. However, *de-novo *algorithms are most useful when spectra have complete or nearly complete fragment peaks and less noisy peaks, because these algorithms rely on the presence of successive *b*- or *y*- ions to find a whole peptide sequence or a sequence tag. *De-novo *algorithms may find ambiguous sequence for real-world spectra because many spectra are far from complete. On the other hand, if a database of all proteins from a genome is accessible, spectra can be assigned to peptides by searching the peptides in the database [1]. Database search based algorithms are currently the leading peptide identification methods. Most database search approaches employ a score function. Different search engines such as Sequest [3] and Mascot [4] adopt different scoring systems. Experiments show that only one search engine may not be reliable for the identification of peptides. To increase the reliability of peptide identifications, some researchers have combined the results of different search engines to assign peptides to spectra. For example, the program Scaffold [5] assigns probabilities to the search results from different peptide identification algorithms such as Mascot [4], Sequest [3], X!Tandem [6], Phenyx [7], Spectrum Mill (Agilent Technologies), and OMSSA [8]. By using the above strategy, it is expected to improve the accuracy of peptide identification from *MS/MS *spectra. However, with the steady increase of the database size, more and more peptides similar to the one investigated can be present in the searched database. On the other hand, the spectrum may contain very few signal peaks or weak signal peaks whose intensities are indistinguishable from those of noisy peaks [9].

Spectral pre-processing, which can improve the reliability of assigning peptides to spectra, becomes very important in current proteomics research. Tandem mass spectrum pre-processing aims at processing spectra produced by tandem mass spectrometers to increase the efficiency of subsequent peptide identification from spectra. Five types of pre-processing methods are widely used: spectrum normalization, spectrum clustering, precursor charge determination, spectrum denoising, and spectrum quality assessment [10-23]. It is believed that these pre-processing algorithms increase the number of identified peptides, and improve the reliability of peptide identification from tandem mass spectra. Now, spectral pre-processing has become a critical module in many high throughput data processing pipelines. Both database search and *de-novo *peptide identification algorithms can benefit from these pre-processing methods.

Spectrum denoising methods intend to keep signal peaks (reflecting peptide fragment ions) while removing noisy peaks (not reflecting peptide fragment ions). In a typical tandem mass spectrum, up to 80% peaks are noises [24]. Therefore, it is necessary to apply a spectrum denoising method before assigning peptides to spectra. In fact, most peptide identification algorithms adopt denoising methods as a pre-processing step. For example, PEAKS [25], PepNovo [26] and AUDENS [27] all have their own denoising models. However, there are many ad hoc problems for spectrum denoising issues. Firstly, the property of un-equally spaced *m/z *values of spectra makes it improper to directly use any standard denoising algorithms for traditional signal processing [16]. Secondly, the noises in a spectrum are hardly modeled by a single statistical model. For example, most noisy peaks are in the middle of *m/z *range of a spectrum, and accordingly, far fewer noisy peaks are in the two ends of a spectrum [24]. Besides, the peaks in the middle of *m/z *range tend to have higher intensities than those at the two ends.

Generally, there exist three types of spectrum denoising algorithms: threshold, digital signal processing, and machine learning or heuristic search algorithms. Threshold methods simply discard peaks with intensities below a threshold. However, the thresholds are hardly determined because a global optimal threshold may not exist for an algorithm to work well. Besides, these methods only use the intensity information of each peak to determine whether a peak is a fragment ion or a noisy peak. These methods implicitly assume the independence of peaks without considering the interrelationship. In fact, a fragment ion may be related to other fragment ions in a true tandem mass spectrum. For example, the mass difference of two signal ions may be equal to the mass of one of the 20 amino acids, e.g., *b*_*i*_, *b*_*i*+1 _ions. The second type of methods uses digital signal processing methods such as Fourier analysis and wavelet analysis for denoising spectra [28]. Digital signal processing methods are successfully used in other fields such as speech recognition, image processing, and computer vision. However, these methods assume that the *m/z *difference between peaks is a constant (interpolation is used to produce equally spaced *m/z *values at the expense of introducing extra peaks). Besides, as the noises are *m/z *dependent, short time Fourier transform or wavelet transform are better choices than Fourier transform. These methods reduce the intensities of the "noisy" peaks without removing them. Just as threshold methods, digital signal processing methods also use the intensity information only. The third type of methods is based on machine learning or some heuristic search using not only intensity information of peaks but also some additional information contained in a spectrum such as isotopic ions or complementary ions [29,30]. However, noises are neither equally distributed in the whole *m/z *range of a spectrum, nor equally distributed among features extracted from a spectrum used for machine learning. As a result, the noises may degenerate the performance of classifiers, and this type of methods may not perform as well as expected. Therefore, we need novel denoising algorithms which are more robust than threshold methods, do not need to introduce extra pseudo peaks, and are "adaptive" to the *m/z *dependence properties of noises in a spectrum.

In this paper, we present a spectral denoising algorithm which partially solves the above mentioned shortcomings of previous denoising algorithms. The proposed algorithm first adjusts the intensities of the peaks of a spectrum using several features extracted from the spectrum. Then the algorithm removes the fragment ions whose intensities are not the local maxima of the intensity-adjusted spectrum using a morphological reconstruction filter [31]. Experiments are conducted on two ion trap mass spectral datasets, and the results show that our algorithm can remove about 69% of the peaks which are likely noisy peaks among a spectrum. At the same time, the number of spectra that can be identified by Mascot increases by 31.23% and 14.12% for the spectra from two datasets.

## Results and Discussion

Similar to [9,16,30], the Mascot search engine is used to evaluate our denoising algorithm. The raw spectra (un-denoised spectra) and the denoised spectra are searched using the Mascot search engine with the same parameters. The parameters used are given in Table 1. A spectrum is identified if its Mascot ion score is larger than a certain threshold. Mascot can provide two thresholds for each peptide: the homology threshold and the identity threshold [32-34] (Note: one can find both the identity threshold and homology threshold for a spectrum by putting the cursor above the query number of the Mascot search report). Each of these two thresholds is different for different peptides. Most proteomics laboratories [32] use the identity threshold as the cut-off value to expect that the false discover rate of the peptide identification is less than (typically) 5%. In this study, we also adopt the identity threshold as the cut-off value, i.e., a spectrum is identified if its Mascot ion score is larger than its identity threshold. By doing so, the false discovery rate is expected to be less than 5% for peptide identification from both the raw and denoised spectra.

**Table 1:** The parameters of Mascot search engine

enzyme	trypsin
fixed modifications	carbamidomethyl
variable modifications	oxidation(M)
peptide charges	+1, +2, +3
mass values	monoisotopic
protein	unrestricted
peptide mass tolerance	± 2*Da*
fragment mass tolerance	± 0.8*Da*
max.missed cleavages	1

### Overall spectrum denoising results

**Figure 1:**
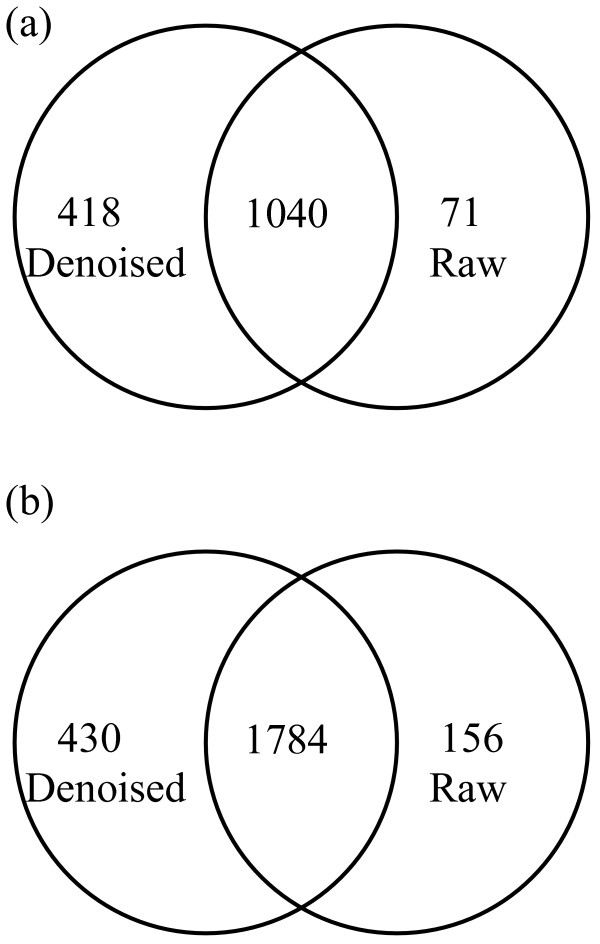
**Venn diagram showing the overlap between the identified spectra from the raw spectra and denoised spectra of *ISB *dataset (a), and *TOV *dataset (b)**.

Experiments are conducted on two ion trap tandem mass spectral datasets (*ISB *and *TOV*) to illustrate the performance of the proposed spectral denoising method by comparing the Mascot search results from the raw datasets to those from the same datasets denoised by the proposed method. The detail description of these two datasets can be found in Section "Material and Methods". The results of comparisons follow as:

Table 2 lists the overall results of experiments. From Table 2, the proposed denoising algorithm can remove about 68.59% (= (156 *- *49)/156) of peaks among a spectrum from *ISB *dataset, and about 68.64% (= (118 *- *37)/118) of peaks among a spectrum from *TOV *dataset. These removed peaks are likely noisy peaks because Mascot performs better after these peaks are removed as discussed below. This study also records the rough Mascot search time (in minutes). From Table 2, by using the proposed denoising algorithm about 13.04% (= (23 *- *20)/23) of search time is saved for the spectra of *ISB *dataset, while about 7.14% (= (14 *- *13)/14) of search time is saved for the spectra of *TOV *dataset. The results illustrate that the proposed method can reduce the time for the process of assigning peptides to spectra because most noisy peaks of a spectrum are removed, especially when the number of spectra in a dataset is large. The number of identified peptides is increased by applying the proposed denoisng method. In Table 2, the number of identified spectra increases by 31.23% (= (1458 *- *1111)/1111) for the spectra of the *ISB *dataset, and 14.12% (= (2214 *- *1940)/1940) for the spectra of the *TOV *dataset. The increasing rate of the newly identified spectra after applying the proposed denoising method is greater for the spectra in *ISB *dataset than for the spectra in *TOV *dataset. The first reason may be that the spectra in *ISB *dataset have more noisy peaks than those in *TOV *dataset. For example, the mean of the number of peaks for the spectra in *ISB *dataset is 156 while that is only 118 for the spectra in *TOV *dataset. The second reason may be that the "quality" of the spectra in *ISB *dataset is inferior to the quality of the spectra in *TOV *dataset. There are 37,044 spectra in *ISB *dataset, but only 1111 spectra (i.e. ~3%) can be identified before applying the proposed denoising method. On the other hand, there are 22,576 spectra in *TOV *dataset, while 1940 (~9%) spectra can be identified before applying the denoising method by Mascot search engine. In addition, from Figure 1(a), up to 93.61% (= 1040/(1040 + 71)) of spectra identified in the raw spectra are also identified after applying the denoising algorithm for *ISB *dataset. Figure 1(b) shows up to 91.96% (= 1784/(1784 + 156)) of spectra identified in the raw spectra are also identified after applying the denoising algorithm for *TOV *dataset.

**Table 2:** The overall results of the denoising algorithm.

Datasets	Mean peaks	Identified	Time
*ISB*			
Raw	156	1111	23
Denoised	49	1458	20

*TOV*			
Raw	118	1940	14
Denoised	37	2214	13

The "Raw" spectra are the original undenoised spectra, and "Denoised" spectra are the denoised spectra. The "Mean peaks" is the mean of the number of peaks of spectra in the dataset; and "Identified" is the number of spectra whose ion scores are greater or equal to the Mascot identity threshold. "Time" is the Mascot search time used in minutes.

We compute the false negative rate of peptide identifications from the *ISB *dataset because these spectra are "standard" spectra, and were intensively studied by other groups [35,36]. Note that the spectra in *ISB *dataset are from 18 known proteins. Thus a spectrum is a false negative if its Mascot ion score is less than its identity threshold while the spectrum is identified from the 18 known proteins by other methods. A spectrum is a false positive if its Mascot ion score is greater than its identity threshold while the spectrum is not identified from the 18 known proteins. Combined the results from [35,36] and our manual verification, we create Table 3 to show distribution of the false positives, and true positives for the denoised spectra and raw spectra. From Table 3, 406 spectra not identified from the raw spectra are false negative for peptide identification, which results in a false negative rate of 26.96% (= 406/1506) for the raw spectral identification. Similarly, 65 spectra not identified from the denoised spectra are false negative for peptide identification, which results in a false negative rate of 4.32% (= 65/1506). In other words, the false negative rate is dramatically reduced from 26.96% to 4.32% after the proposed algorithm is applied. This indicates that Mascot can perform much better by combining with the proposed method, given the same false discovery rate of 5% controlled by the Mascot identity threshold.

**Table 3:** The distributions of the false positives and true positives in *ISB *spectra identified by the Mascot search engine.

	Denoised	Overlap	Raw	Total
*FP*	12	5	6	23
*TP*	406	1035	65	1506

Total	418	1040	71	1529

The "Denoised" spectra can be identified only after denoising. The "Overlap" spectra can be identified from both the denoised and the raw spectra. The "Raw" spectra can be found only in the original undenoised spectra. "Total" counts the sums. "*FP *" are the false positives in the identified spectra, and "*TP *" are the true positives in the identified spectra.

### The functions of each module

**Figure 2:**
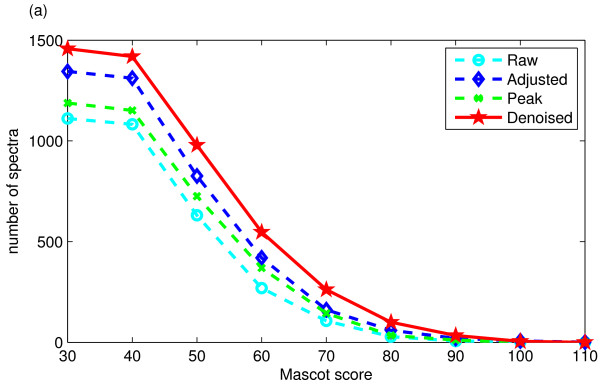
**The number of spectra whose Mascot ion scores are greater than a given value for the raw and the processed spectra in *ISB *dataset (a) and *TOV *dataset (b)**. Here the "Raw" spectra are the unprocessed spectra; "Adjusted" spectra are the peak intensity-adjusted spectra; "Peak" spectra are the spectra processed by the morphological filter; and "Denoised" spectra are the spectra processed by peak extraction after intensity adjustment.

The proposed algorithm has two modules: intensity adjustment and peak extraction. The functions of each module in the proposed algorithm are investigated in terms of peptide ion scores. As shown in Figure 2, both intensity adjustment and peak extraction can increase the number of identified spectra, but peak extraction combined with intensity adjustment can help to identify more spectra than using either an individual module.

### Discussion and further improvement

Our proposed algorithm does not need to resample each spectrum to have the same *m/z *distance between two adjacent peaks. Therefore, the algorithm neither introduces additional "noisy" peaks nor changes the *m/z *of each peak. This property is one of the advantages of our algorithm over other denoising algorithms based on Fourier analysis and wavelet analysis, e.g., MS-cleaner [16].

Unlike threshold based methods, our algorithm does not need to provide a global threshold. In fact, the morphological reconstruction filter can be considered as an adaptive signal processing method, as it "adaptively" extracts the local maxima of a spectrum. This property of morphological reconstruction filter indicates that our algorithm could be more robust than threshold based denoising algorithms [25].

In the proposed algorithm, for the intensity adjustment module, the values of the parameters are chosen according to Sequest, and these values are proved to be effective in identifying peptides from spectra. For the morphological reconstruction filter, there is only one parameter to choose. This parameter can be set as a very small value, e.g., the smallest intensity difference between two peaks. While for the methods based on wavelet analysis, one needs to choose several parameters such as the wavelet basis functions and the thresholds of the wavelet coefficients. These parameters can significantly influence the final denoising results.

The proposed algorithm uses more information about a theoretical peptide fragment ion in denoising spectra. We construct several features to adjust the intensities of a peak. Although the intensities of peaks at the two ends of each spectrum are less enhanced than those in the middle of *m/z *range, the intensities of signal peaks are still enhanced more than those of the noisy peaks. Thus the signal peaks are still local maxima of a spectrum, and the morphological reconstruction filter can correctly discriminate the signal peaks from noisy ones. From this point of view, our method is more robust than machine learning based denoising algorithms [30] because our algorithm decreases the influence of the unequally distributed noises in tandem mass spectra.

The influence of the denoising method is different to the spectra with different charge states. As shown in Table 4, Mascot can identify another 177 triply charged spectra in *ISB *dataset after applying the proposed denoising algorithm, i.e., about 42.34% (= 177/418) of newly identified spectra are triply charged. The number of triply charged spectra accounts for about 33.80% (= 24/71) of the lost spectra. Therefore the denoising algorithm can help to find more triply charged spectra. This phenomenon is more obvious for the spectra in *TOV *dataset. For example, about 24.88% (= 107/430) of newly identified spectra are triply charged, while only 12.82% (= 20/156) of spectra are triply charged of all the lost spectra after applying the denoising algorithm. While for singly charged spectra, although the denoising method can increase the number of identified spectra, the singly charged spectra account for about 15.49% (= 11/71) of the lost spectra. This number is relatively large taking into consideration the small number of originally identified singly charged spectra. Therefore, one can expect that a denoising algorithm which employs several properties of a tandem mass spectrum (such as its charge state and the number of peaks [30]) performs better than the one which employs a single property of the tandem mass spectrum. The proposed denoising algorithm can be tuned to preprocess tandem mass spectra for other peptide identification algorithms such as Sequest or de-novo algorithms. Note that Sequest algorithm is based on convolution technique. The convolution results are determined by the peaks which have extra-large intensities even if experimental spectra are normalized first in Sequest algorithm. For this reason, we may need to design other spectral normalization algorithms [18,37] or change the intensities of peaks which are not removed after applying the denoising algorithm back to their original intensities. Anyway, because noisy peaks are removed, peptide identification algorithms can benefit from the proposed denoising algorithm. But for specific peptide identification algorithms, because their different use of intensity information of spectra, specific normalization algorithms are needed for these algorithms to work optimally. A further improvement of the proposed denoising algorithm is to combine denoising algorithms with quality assessment algorithms for pre-processing tandem mass spectra. By this way, we can improve the reliability of assigning peptides to spectra, and increase the information that can be extracted from tandem mass spectra. For example, if the features used for enhancing intensities of peaks of a spectrum are very small, this spectrum may be a poor quality spectrum, and this spectrum can be excluded from further processing.

**Table 4:** The influence of charge states to the filtering results.

Datasets	Single	double	triple	Total
*ISB*				
New	20	221	177	418
Overlap	12	695	333	1040
Lost	11	36	24	71

*TOV*				
New	14	309	107	430
Overlap	12	1638	134	1784
Lost	5	131	20	156

Here "Single", "Double" and "Triple" represent different charge states. The "New" spectra are the newly identified spectra after denoising. The "Overlap" spectra can be identified from both the denoised and the raw spectra. The "Lost" spectra are lost after denoising.

## Conclusion

This paper has presented a spectral denoising algorithm. The proposed algorithm first adjusts the intensities of spectra. After peak intensity adjustment, the intensities of signal peaks in a spectrum become local maxima of the spectrum. Second, the peak intensity-adjusted spectra are filtered using a morphological reconstruction filter. The signal peaks are kept while the noisy peaks are removed after applying the morphological reconstruction filter. By applying the denoising method, about 69% of peaks among a spectrum can be removed. At the same time, the number of spectra that can be identified by Mascot algorithm increases by 31.23% and 14.12% for the spectra in *ISB *dataset and *TOV *dataset, respectively. In summary, the proposed algorithm can remove most of noisy peaks, and increase the reliability of assigning peptides to spectra. As a result, more peptides can be identified from denoised spectra than from raw spectra.

## Materials and methods

The proposed spectral denoising method consists of two unique modules: peak intensity adjustment and intensity local maximum extraction. The first one is used to adjust the intensities of signal peaks in a spectrum. After adjustment, intensities of signal peaks are expected to be the local maxima of the spectrum. The second one is used to select these local maxima of the signal peak intensity-adjusted spectra, and thus peaks whose intensities are not the local maxima are removed.

### Datasets

This study employs two ion trap tandem mass spectral datasets: *ISB *dataset and *TOV *dataset to investigate the performance of the introduced denoising algorithm. The following is a brief description of these datasets.

#### (1) ISB dataset

The spectra in *ISB *dataset are acquired from a low resolution *ESI *ion trap mass spectrometer as described in [35]. These spectra consisting of 22 LC/MS/MS runs are produced by Institute of System Biology (*ISB*) from 18 control mixture proteins. There are a total of 37, 044 spectra in *ISB *dataset. These spectra are searched using Mascot against the ipi.HUMAN.v3.48.fasta (taken from EMBL-EBI, ) containing 71, 399 sequences and 5 contaminant sequences (P00760, P00761, P02769, Q29443 and Q29463 from ) appended with the sequences of the mixture proteins (from ).

#### (2) TOV dataset

The *MS/MS *spectra are acquired from a LCQ DECA XP ion trap spectrometer (ThermoElectron Corp.) as described in [38]. The number of spectra in this dataset is 22, 576, and these spectra are searched using Mascot against the ipi.HUMAN.v3.42.fasta (taken from EMBL-EBI, ) containing 72, 340 protein sequences and 5 contaminant sequences (P00760, P00761, P02769, Q29443 and Q29463 from ).

### Peak intensity adjustment

The intensity is an important attribute of a peak in a spectrum. The empirical approaches usually assume that peaks with high intensities are more likely to be signal peaks than those with low intensities.

However, there are many exceptions to these approaches. Thus to distinguish signal peaks from noisy peaks, more attributes of peaks should be taken into consideration. For example, signal peaks may have complementary peaks whose masses are added to the signal peaks to give the mass of a precursor ion. In this study, five features are constructed for each peak on basis of the properties of theoretical peptide mass spectra [22]. A score for each peak is calculated by a linear combination of these features. To define these features, as in [22], four variables are firstly introduced

$$\begin{array}{c} dif1(x,y)=x-y\\ dif2(x,y)=x-(y+1)/2\\ sum1(x,y)=x+y\\ sum2(x,y)=x+(y+1)/2 \end{array}$$

For a peak with the *m/z *value of *x *(for simplicity, this peak is called peak *x*) of a spectrum *S*, the first feature *F*_1 _collects the number of peaks whose mass differences with *x *approximately equal the mass of one of the twenty amino acids.

$$\begin{array}{l} F_{1}=|\{y|abs(dif1(x,y))\approx M_{i}\text{ or}\\ abs(dif1(x,y))\approx M_{i}/2\text{ or}\\ abs(dif2(x,y))\approx M_{i}/2\text{ or}\\ abs(dif2(y,x))\approx M_{i}/2\}| \end{array}$$

where |•| is the cardinality of a set; *y *is the *m/z *value of a peak in *S*; *abs *is the absolute value function; and *M*_*i*_(*i *= 1, 2,..., 20) is the mass of one of the twenty amino acids. In this study we consider all Methionine amino acids to be sulfoxidized and do not distinguish three pairs of amino acids in their masses: Isoleucine vs. Leucine, Glutamine vs. Lysine, and sulfoxidized Methionine vs. Phenylalanine as the masses of each pair are very close. If both peaks *x *and *y *are singly charged, their difference equals the mass of one of the 20 amino acids, and *abs*(*dif*1(*x*, *y*)) ≈ *M*_*i*_; if both *x *and *y *are doubly charged, their difference equals half of the mass of one of the 20 amino acid, and *abs*(*dif*1(*x*, *y*)) ≈ *M*_*i*_/2; if *x *is singly charged while *y *is doubly charged, *abs*(*dif*2(*x*, *y*)) equals half of one of the mass of the 20 amino acids; and if *x *is doubly charged while *y *is singly charged, *abs*(*dif*2(*y*, *x*)) equals half of the mass of one of the 20 amino acids. The comparison implied by ≈ uses a tolerance. Bern *et al *used ± 0.37 [18] for constructing features for the quality assessment of ion trap tandem mass spectra. Wong *et al *used ± 0.3 for fragment ion mass tolerance, and ± 1 for precursor ion mass tolerance for ion trap tandem mass spectra [39]. In this study, we use ± 0.8 for fragment ion mass tolerance, and ± 2 for precursor ion mass tolerance because these parameters are reasonable for ion trap spectra for the Mascot search engine to give good peptide identification results.

The second feature *F*_2 _collects the number of peaks whose masses added to *x *approximately equal the mass of the precursor ion.

$$\begin{array}{l} F_{2}=|\{y|sum1(x,y)\approx M_{parent}+2*m(H)\text{ or}\\ sum1(x,y)\approx M_{parent}/2+2*m(H)\text{ or}\\ sum2(x,y)\approx M_{parnet}/2+2*m(H)\text{ or}\\ sum2(y,x)\approx M_{parnet}/2+2*m(H)\}| \end{array}$$

where *M*_*parent *_is the mass of the precursor ion (parent). As for *F*_1_, if both peaks *x *and *y *are singly charged, *sum*1(*x*, *y*) ≈ *M*_*parent *_+ 2 * m(*H) *; if both *x *and *y *are doubly charged, *sum*1(*x*, *y*) ≈ *M*_*parent*_/2 + 2 * m(*H*); if *x *is singly charged while *y *is doubly charged, *sum*2(*x*, *y*) ≈ *M*_*parnet*_/2 + 2 * m(*H*); and if *x *is doubly charged while *y *is singly charged, *sum*2(*y*, *x*) ≈ *M*_*parnet*_/2 + 2 * m(*H*).

The third feature *F*_3 _collects the number of peaks which are produced by losing a water or an ammonia molecule from *x*.

$$\begin{array}{l} F_{3}=|\{y|dif1(x,y)\approx M_{water}\text{ or }M_{ammonia}\text{ or}\\ dif1(x,y)\approx M_{water}\text{/2 or }M_{ammonia}\text{/2 or}\\ dif2(x,y)\approx M_{water}\text{/2 or }M_{ammonia}\text{/2 or}\\ -dif2(y,x)\approx M_{water}\text{/2 or }M_{ammonia}\text{/2\}|} \end{array}$$

where *M*_*water *_is the mass of a water molecule and *M*_*ammonia *_is the mass of an ammonia molecule. Because *x *loses a molecule to form *y*, *x *should be larger than *y *if they have the same charge state. Therefore, different from *F*_1_, the *abs *function should not be used here. If both peaks *x *and *y *are singly charged, *dif*1(*x*, *y*) ≈ *M*_*water *_or *M*_*ammonia*_; if both *x *and *y *are doubly charged, *dif*1(*x*, *y*) ≈ *M*_*water*_/2 or *M*_*ammonia*_/2; if *x *is singly charged while *y *is doubly charged, *dif*2(*x*, *y*) ≈ *M*_*water*_/2 or *M*_*ammonia*_/2; and if *x *is doubly charged while *y *is singly charged, a minus sign should be added to *dif*2(*y*, *x*) and *-dif*2(*y*, *x*) ≈ *M*_*water*_/2 or *M*_*ammonia*_/2.

The fourth feature collects the number of peaks which are produced by losing a *CO *group or an *NH *group from *x*.

$$\begin{array}{l} F_{4}=|\{y|dif1(x,y)\approx M_{CO}\text{ or }M_{NH}\text{ or}\\ dif1(x,y)\approx M_{CO}\text{/2 or }M_{NH}\text{/2 or}\\ dif2(x,y)\approx M_{CO}\text{/2 or }M_{NH}\text{/2 or}\\ -dif2(y,x)\approx M_{CO}\text{/2 or }M_{NH}\text{/2\}|} \end{array}$$

where *M*_*CO *_and *M*_*NH *_are the mass of a *CO *group and an *NH *group, respectively. For the same reason as for *F*_3_, *x *should be larger than *y *if they have the same charge state. Therefore, if both peaks *x *and *y *are singly charged, *dif*1(*x*, *y*) ≈ *M*_*CO *_or *M*_*NH *_; if both *x *and *y *are doubly charged, *dif*1(*x*, *y*) ≈ *M*_*CO*_/2 or *M*_*NH*_/2; if *x *is singly charged while *y *is doubly charged, *dif*2(*x*, *y*) ≈ *M*_*CO*_/2 or *M*_*NH*_/2; and if *x *is doubly charged while *y *is singly charged, the two peaks should satisfy *-dif*2(*y*, *x*) ≈ *M*_*CO*_/2 or *M*_*NH*_/2. The fifth feature is used to collect the number of isotope peaks associated with *x*

*F*_5 _= |{*y|M*(*x*) ≈ *M*(*y*) *- *1 or *M*(*x*) ≈ *M*(*y*) *- *0.5)}|

The adjusted intensity of each peak is the original intensity of the peak multiplied by the score computed on basis of the five features. The final score of each peak is calculated as:

*Score *= *ω*_0 _+ *ω*_1 _* f_1 _+ *ω*_2 _* f_2 _+ *ω*_3 _* f_3 _+ *ω*_4 _* f_4 _+ *ω*_5 _* f_5_

where *f*_*i*_(*i *= 1,..., 5) is the normalized value of each feature (Normalized to have the mean of zero and the variance of one), and *ω*_*i*_(*i *= 0,..., 5) are the coefficients. This study sets the bias *ω*_0 _= 5 to ensure only few peaks have negative score; *ω*_1 _and *ω*_2 _are set to 1.0; both *ω*_3 _and *ω*_4 _are set to 0.2; and *ω*_5 _is set to 0.5.

These values are selected according to the normalization method of Sequest algorithm. In Sequest algorithm, a magnitude of 50 is assigned to the *b*- and *y*- ions in a theoretical spectrum. The neutral loss of water ions, the neutral loss of ammonia ions, and *a*- ions are assigned a value of 10. The ions which have mass difference of ± 1 with *b*- and *y*- ions are assigned a value of 25. In this study the values are slightly different from those of the Sequest algorithm to avoid numerical problems incurred by multiplying large numbers, but the relative importance of the value of each parameter is the same as the value of the corresponding parameter of Sequest search engine. Note that the Sequest algorithm does not consider complementary ions. However, from the study of other peptide identification algorithms such as Mascot and our own study, the complement ions are very likely to be signal peaks, e.g., the presence of complementary ions is a very important feature to predict whether a spectrum is of high or poor quality [22,37]. Therefore, the weight value for feature *F*2 is assigned the same as that for feature *F*_1_. The score function is similar to linear discriminative analysis (*LDA*) which combines a finite number of features into a score.

This study does not use these features to train a classifier to classify a peak as a signal peak or a noisy peak because of the peak distribution properties of tandem mass spectra. For example, the number of peaks in the middle of *m/z *value range of a spectrum is larger than the number of peaks in the two ends of the spectrum, and most noisy peaks are in the middle of *m/z *value range. Thus the features we constructed are *m/z *dependent. In addition, the masses of peptides are widely scattered, and the number of peaks of spectra are quite different. These are all challenges for machine learning algorithms. Elaborated normalization methods are necessary before using these algorithms.

The intensities of signal peaks are increased while the intensities of noisy peaks are decreased after peak intensity adjustment. However, simple threshold is still not effective to differentiate signal peaks from noisy ones because the scores of peaks in a spectrum tend to be larger in the middle of *m/z *range than the scores of the two ends peaks because most noisy peaks are in the middle of *m/z *range of a spectrum. It is more reasonable to assume that the noisy peaks in a narrow *m/z *range are equally distributed, and that the signal peaks are mostly the local maxima of a spectrum after peak intensity adjustment. Therefore, noisy peaks can be removed by keeping only these local maxima.

### Peak local maximum extraction

**Figure 3:**
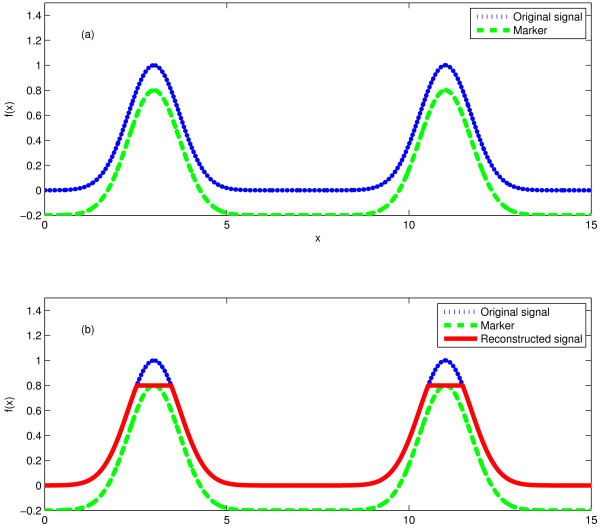
**An example of morphological reconstruction filter**. The "marker" is obtained by subtracting a small value of 0.2 from the original signal (a), and the difference between the original signal and the reconstructed signal corresponds to the local maxima of the original signal (b).

This study employs an algorithm called morphological reconstruction filter [31] to select the local maxima of a spectrum. The inputs of a morphological reconstruction filter are a "mask" signal which is the original signal, and a "marker" signal which specifies the preserved parts in the reconstructed signal. In this study, a mask signal is a tandem mass spectrum while its marker signal is the mask signal subtracted by a very small positive number of 0.0001. Morphological reconstruction filter can be considered as repeated dilations of the marker signal until the contour of the dilated marker signal fits under the mask signal [31,40]. In each dilation the value of the marker signal at every point will take the maximum value over its neighborhood. As a result, the values of the dilated marker signal are increased except the local maxima of the marker signal which will stay the same as before. The dilation operation is constrained to lie underneath the mask signal. When further dilations do not change the marker signal any more, the process stops. At this point, the dilated marker signal is exactly the same as the mask signal except the local maxima. By comparing the mask signal and the dilated marker signal, the local maxima of the mask signal can be extracted. Figure 3 shows an example of morphological reconstruction filter to extract the local maxima.

## Competing interests

The authors declare that they have no competing interests.

## Authors' contributions

JD developed the algorithm, designed and executed all experimental work, and wrote the first draft. JHS participated in the discussion and the modification of the manuscript. FXW supervised and initiated project, and intensively revised the manuscript. GGP carried out the collection of *TOV *data, participated in interpretation of data analysis and modification of the manuscript. All authors read and approved the manuscript.
